# Risk factors, microbiological findings and outcomes of necrotizing fasciitis in New Zealand: a retrospective chart review

**DOI:** 10.1186/1471-2334-12-348

**Published:** 2012-12-12

**Authors:** Dilip Kumar Das, Michael G Baker, Kamalesh Venugopal

**Affiliations:** 1 Department of Public Health, University of Otago, Wellington, New Zealand; 2 Regional Public Health, Hutt Valley District Health Board, Private Bag 31 907, Lower Hutt, 5040, New Zealand

**Keywords:** Bacterial infection, Ethnicity, Necrotizing fasciitis, New Zealand, Traditional Samoan tattooing

## Abstract

**Background:**

The incidence and mortality from necrotizing fasciitis (NF) are increasing in New Zealand (NZ). Triggered by a media report that traditional Samoan tattooing was causing NF, we conducted a chart review to investigate the role of this and other predisposing and precipitating factors and to document NF microbiology, complications and interventions in NZ.

**Methods:**

We conducted a retrospective review of 299 hospital charts of patients discharged with NF diagnosis codes in eight hospitals in NZ between 2000 and 2006. We documented and compared by ethnicity the prevalence of predisposing and precipitating conditions, bacteria isolated, complications and interventions used.

**Results:**

Out of 299 charts, 247 fulfilled the case definition. NF was most common in elderly males. Diabetes was the most frequent co-morbid condition, followed by obesity. Nearly a quarter of patients were taking non-steroidal anti-inflammatory drugs (NSAID). Traditional Samoan tattooing was an uncommon cause. *Streptococcus pyogenes* and *Staphylococcus aureus* were the two commonly isolated bacteria. Methicillin-resistant *Staphylococcus aureus* was implicated in a relatively small number of cases*.* Shock, renal failure, coagulation abnormality and multi-organ dysfunction were common complications. More than 90% of patients underwent surgical debridement, 56% were admitted to an intensive care unit (ICU) and slightly less than half of all patients had blood product transfusion. One in six NF cases had amputations and 23.5% died.

**Conclusion:**

This chart review found that the highest proportion of NF cases was elderly males with co-morbidities, particularly diabetes and obesity. Tattooing was an uncommon precipitating event. The role of NSAID needs further exploration. NF is a serious disease with severe complications, high case fatality and considerable use of health care resources.

## Background

Necrotizing fasciitis (NF) is a rapidly progressive soft tissue infection characterized by necrosis of the subcutaneous tissue and fascia [1,2]. Although rare, NF frequently causes severe illness resulting in death or permanent disability [2]. Analysis of hospitalization data in New Zealand (NZ) for the period 1990 to 2006 showed that incidence and mortality rates of NF have increased significantly and the incidence is significantly higher in Pacific people and Maori than in Europeans and people of Other ethnicity [3]. The causes of the increase and differential ethnic distribution in incidence are not known [3].

The pathogenesis of NF involves a complex interplay between agent and host factors. Most patients who develop NF have pre-existing conditions that render them susceptible to infection [4], although it also occurs in young, previously healthy individuals [2,5]. The disease occurs more frequently in the elderly [4,6,7], diabetics [4,6-10], alcoholics [6-8], intravenous drug users [2,4,6,11], in patients with chronic liver disease [2], renal insufficiency [4,7,8], peripheral vascular disease [4,6,7], gout [8,12], underlying malignancy [4,6,7] or immunocompromised states [4,6]. Other factors which have been found to be associated with NF are obesity [4,6], malnutrition [4,6], chronic obstructive pulmonary diseases [7,9] and congestive heart failure [9]. Many NF patients are found to be taking non-steroidal anti-inflammatory drugs (NSAID) at the time of presentation to hospital [9,12,13], but its role remains unclear [14,15].

Usually the disease is precipitated by some form of injury or local pathological condition [7]. Blunt or penetrating trauma [4], operation site infection [4], burn [4], ulcers [16], abscess [16] and even child birth [4] have been documented as precipitating factors for NF. Body piercing procedures such as acupuncture [6] and tattooing [17], very minor trauma such as abrasion [16] and insect bites [16] have also been found to initiate NF. In some situations NF has arisen without identifiable antecedent trauma [7,16] or pathological state [7].

Tattooing and skin piercing are invasive procedures and can have infective complications if not done in a sterile way [18,19]. A few cases of NF and severe soft tissue infection following traditional Samoan tattooing have been reported in NZ [17,20] drawing wide media attention [21].

NF may be caused by a variety of aerobic and facultative anaerobic bacteria, including *Streptococcus pyogenes* or group A streptococci (GAS), *Staphylococcus aureus*, *Escherichia coli*, Clostridium and Bacteroides species [1]. Rarely, group B, C, and G streptococci, *Haemophilus influenzae type b*, *Pseudomonas aeruginosa*, *Vibrio vulnificus*, and fungi are involved [1]. Frequently the disease is polymicrobial [16,22]. The microbiology of NF in NZ has not been well documented.

Managing NF uses a large amount of health-care resources [23]. It can also cause serious complications [23], such as acute renal failure, shock, multi-organ dysfunction, coagulopathy, arrhythmia, and myocardial infarction. Its management requires intensive care unit (ICU) admission in many cases, and urgent surgical debridement and intravenous antibiotics in almost all cases [1,2,24]. Many patients who survive require reconstructive surgery and skin grafting (often with multiple admissions) [25]. Blood product transfusion and dialysis are also needed to manage specific complications. The disease is associated with a high case fatality risk and permanent disability in the form of limb loss [26].

We conducted a retrospective chart review to investigate the relationship between the incidence of NF in NZ and traditional Samoan tattooing and other risk factors, and to document the microbiology, complications, treatment and outcomes of NF. A particular focus was to compare these characteristics across ethnic groups.

## Methods

To explore the role of traditional Samoan tattooing in NF, we purposely selected hospitals with NF patients of Pacific ethnicity for chart review. From Ministry of Health hospital discharge data for the period 2000–2006, we identified hospitals where at least one person of Pacific ethnicity was admitted with NF during this period. These hospitals were: Auckland City, Starship, North Shore, Middlemore, Tauranga, Rotorua, Hutt and Wellington. These hospitals included 58% of the 513 cases discharged from NZ hospitals with an NF diagnosis over that seven year period.

By using the International Classification of Diseases (ICD) codes of NF (M7250 to M7269 in ICD-10-AM versions 1, 2 and 3) we identified the National Health Index (NHI) numbers of all NF patients (all ethnicities) in these hospitals admitted between 2000 and 2006 and subsequently located their charts.

One investigator (DD) visited Medical Records Departments of the above mentioned hospitals and extracted relevant data from patients’ charts and entered these into a Microsoft Access database.

A case was included in the chart review if it satisfied the following criteria:

Discharge diagnosis of NF (M7250 – M7269 in ICD-10), irrespective of whether NF was the principal diagnosis or an additional diagnosis; AND

Operation notes clearly indicated presence of necrosis in the fascia and subcutaneous tissue; OR

Operative finding specifically mentioned ‘necrotizing fasciitis’; OR

Histopathology of debrided fascia and subcutaneous tissue or autopsy findings of local tissue showed necrosis.

If the case has been coded as ‘NF’ or as ‘Fasciitis, Not Elsewhere Classified,’ but the operative or histopathology or autopsy criteria were not satisfied, the chart was not reviewed further.

We checked the data for any inconsistencies and outliers and corrected these in the data preparation stage. Frequency tables of variables of interest were generated. Tests of statistical significance were done using the Chi-square test. We used level 1 prioritized ethnicity and four ethnic groupings: European, Maori, Pacific and Other (including Asian) [27]. Data were analyzed using Statistical Package for the Social Sciences for Windows version 9 (SPSS Inc., Chicago, IL) and Epi Info™ version 6 (Centers for Disease Control and Prevention, Atlanta, GA).

Multi-Region Ethics Committee approval was obtained to access, review and extract the relevant data from NF patients’ charts from the selected hospitals.

## Results and discussion

### Patients included

We reviewed 299 NF patient charts of which 247 (82.6%) satisfied the case inclusion criteria. Conditions like diabetic gangrene of toe, foot and leg, cellulitis, abscess, plantar fasciitis, Dupuytren’s contracture, and limb loss due to invasive meningococcal disease were sometimes coded as fasciitis or NF. However, these were clearly not cases of NF and were excluded from the analysis. Operative and histology findings criteria were the two most commonly used criteria for assigning disease status. However, for more than half of the charts a histology report was not available. Many operative notes did not mention specific operative findings but just mentioned ‘NF found at operation’. Consequently, a very tight case definition could not always be applied.

### Demography of reviewed patients

Table 1 shows the age, sex and ethnicity distribution of the 247 NF cases fulfilling the case definition. Males comprised 61% and females 39% of the total. Amongst Europeans the highest number of cases was in the 70+ age group, while for Maori and people of Pacific and Other ethnicity, the number of cases was highest in the 50–69 year age group. The median age of European cases (61.4 years) was significantly higher than Maori (48.9 years) and Pacific (50.8 years) (p < 0.001).

**Table 1:** Age, sex and ethnicity distribution of NF patients reviewed

**Age group (years)**	**European (n=122)**		**Maori (n=47)**		**Pacific (n=60)**		**Others (n=17)**		Total* (n=247)	
	**M**	**F**	**M**	**F**	**M**	**F**	**M**	**F**	**M**	**F**
0-14	2	1	1	2	2	1	0	0	5	4
15-29	3	3	5	3	5	3	0	2	13	11
30-49	17	11	11	5	13	5	2	2	43	23
50-69	25	15	10	7	14	11	7	2	56	35
70+	24	21	2	1	5	1	1	1	32	24
Total*	71	51	29	18	39	21	10	7	150	97
(%)	(58.2)	(41.8)	(61.7)	(38.3)	(65.0)	(35.0)	(58.8)	(41.2)	(60.7)	(39.3)

M – Male, F – Female. *Total includes one male case with ‘unknown’ ethnicity.

### Predisposing conditions

Table 2 shows the ethnic distribution of selected predisposing conditions. Diabetes was the most common (32.0%) predisposing condition, followed by obesity (22.7%). Nearly a quarter of the patients (23.1%) were reported to have taken NSAID within the seven days preceding development of NF. About a fifth of the patients did not have any predisposing condition recorded in their charts.

**Table 2:** Presence of known predisposing conditions for NF by ethnicity

**Predisposing conditions**	**European (n=122)**		**Maori (n=47)**		**Pacific (n=60)**		**Others (n=17)**		**Total (n=247)**		**p-value**
	**No.**	**%**	**No.**	**%**	**No.**	**%**	**No.**	**%**	**No.**	**%**	
Diabetes mellitus	28	23.0	14	29.8	27	45.0	10	58.8	79	32.0	0.002
Alcohol abuse	11	9.0	3	6.4	0	0.0	1	5.9	15	6.1	0.126
Obesity	23	18.9	16	34.0	14	23.3	3	17.6	56	22.7	0.193
Coronary heart disease	22	18.0	8	17.0	5	8.3	5	29.4	40	16.2	0.156
Congestive heart failure	15	12.3	6	12.8	10	16.7	2	11.8	33	13.4	0.865
Peripheral vascular disease	14	11.5	6	12.8	4	6.7	2	11.8	26	10.5	0.720
Chronic obstructive	16	13.1	4	8.5	2	3.3	0	0.0	22	8.9	0.086
pulmonary disease											
Cancer	12	9.8	3	6.4	1	1.7	2	11.8	18	7.3	0.210
Chronic renal failure	11	9.0	9	19.1	7	11.7	2	11.8	29	11.7	0.341
Steroid use	14	11.5	2	4.3	1	1.7	0	0.0	17	6.9	0.040
Non-steroidal anti-inflammatory drug use*	29	23.8	11	23.4	10	16.7	7	41.2	57	23.1	0.208
No condition documented	18	14.8	10	21.3	20	33.3	0	0.0	49	19.8	0.004

*Low-dose Aspirin 100mg/day or less excluded.

Some conditions were comparatively more prevalent in particular ethnic groups, e.g., diabetes in Others and Pacific people, alcohol abuse in Europeans, obesity in Maori and Pacific people, coronary heart disease in Others, congestive heart failure in Pacific people, chronic obstructive pulmonary disease (COPD) in Europeans, cancers in Others and Europeans, steroid use in Europeans, NSAID use in Others and chronic renal failure in Maori. However, the ethnic difference in prevalence was significant only for diabetes (p < 0.01) and steroid use (p < 0.05). P-values in this and subsequent tables should be interpreted with caution where some cells have values <5. There was also a statistically significant difference (p 0.004) in the ethnic distribution of cases with ‘no condition documented’ (Table 2). From the available clinical notes it is not possible to say the cause of this association. The significantly greater age of the European cases may have contributed to the higher documented prevalence of co-morbidities for NF cases from that ethnic group.

### Precipitating events

The distribution of precipitating events by ethnicity is shown in Table 3. By far the most commonly reported precipitating event was accidental trauma (33.2%), followed by skin ulcer (15.8%) and surgery (11.3%). The type of trauma ranged from superficial graze to blunt trauma without skin break, laceration and penetrating injuries. This group also included insect and animal bites. Five patients had a history of insect bite (one mosquito, one sand fly and three unspecified insect bites) as precipitating events. One person developed NF following a dog bite on their leg.

**Table 3:** Presence of precipitating events for NF by ethnicity in reviewed patients

**Precipitating events**	**European (n=122)**		**Maori (n=47)**		**Pacific (n=60)**		**Others (n=17)**		**Total (n=247)**		**p-value**
	**No.**	**%**	**No.**	**%**	**No.**	**%**	**No.**	**%**	**No.**	**%**	
Traditional tattooing	0	0.0	0	0.0	4	6.7	0	0.0	4	1.6	0.005
Accidental trauma	43	35.2	15	31.9	19	31.7	4	23.5	82	33.2	0.791
Skin ulcer	20	16.4	9	19.1	5	8.3	4	23.5	39	15.8	0.289
Surgery	16	13.1	6	12.8	4	6.7	2	11.8	28	11.3	0.619
Burns	3	2.5	0	0.0	0	0.0	0	0.0	3	1.2	0.378
Current IVDU*	2	1.6	1	2.1	0	0.0	0	0.0	3	1.2	0.693

*IVDU= Intravenous drug use.

Only four patients (1.6% of the total) developed NF directly following traditional Samoan tattooing. All of them were Samoan and one of them died. Not surprisingly, the p value of this category indicates statistically significant ethnic differences in exposure to this precipitating event (Table 3). Two people (0.8%) were noted to have acupuncture in the affected area before developing NF.

### Microbiology

Out of 247 NF patients’ charts reviewed, either debrided tissue, blood, aspirated fluid or wound swab culture results (in the form of hard copy or electronic laboratory reports) were available for 219. Another 19 patients had their culture results documented in their clinical notes but no laboratory reports were filed in their charts. Specimens were taken at operation (debrided tissue or swab) or preoperatively (blood, blister fluid or wound swab). In total, there were culture positive results from one or more specimens in 96% (228/238) patients. The extent of microbiology investigations and their documentation in different hospitals were not uniform. For example, the number of antibiotics against which a cultured organism was tested for susceptibility differed substantially from hospital to hospital and also within the hospital from case to case. We are not sure whether specimens were cultured for all possible organisms. For example, for many cases anaerobic culture reports were not available. Some reported specific anaerobic organisms isolated while some indicated that there was growth of mixed anaerobes. It is also important to note that a third of all patients received antibiotics before hospital admission and specimens for culture were taken. Table 4 is a list of organisms isolated either solely or in combinations, and their frequency of occurrence.

**Table 4:** Micro-organisms isolated from NF patients for whom culture results were available (n=238)

	**Count**	**Percentage**
Organisms and characteristics:		
· Gram positive cocci		
*Streptococcus pyogenes*	98	41.2%
Other *streptococci spp.*	42	17.6%
*Staphylococcus aureus (SA)*	76	31.9%
Other *staphylococci spp.*	37	15.5%
MRSA	8	3.4%
· Gram positive bacilli		
*Corynebacterium spp.*	5	2.1%
· Mixed gram positive organisms	4	1.7%
· Gram negative bacilli		
*E coli*	23	9.7%
*Proteus spp.*	8	3.4%
*Klebsiella spp.*	3	1.3%
*Enterobacter spp.*	2	0.8%
*Serratia spp.*	5	2.1%
*Citrobacter spp.*	1	0.4%
*Pseudomona spp.*	17	7.1%
*Eikenella spp.*	1	0.4%
*Providencia spp.*	1	0.4%
*Vibrio spp.*	1	0.4%
*Stenotrophomonas spp.*	2	0.8%
*Hafnia spp.*	1	0.4%
*Aeromonas hydrophilia*	1	0.4%
Mixed gram negative bacilli	7	2.9%
· Anaerobes		
*Bacteroides spp.*	11	4.6%
*Prevotella spp*	3	1.3%
*Clostridium spp*	2	0.8%
*Preptostreptococcus spp.*	1	0.4%
Mixed anaerobes	29	12.2%
· Mixed bowel flora	4	1.7%
· Mixed skin flora	7	2.9%
· Fungus		
*Candida spp.*	1	0.4%

The most common organism isolated was *Streptococcus pyogenes* (41.2%) either solely (20.6%) or in combination with other organisms (20.6%). Streptococci other than *Streptococcus pyogenes*, usually in combination with other organisms, were isolated in 17.6% cases. For most of the *Streptococcus pyogenes* isolates antibiotic susceptibility testing was not done. The culture reports mentioned that *Streptococcus pyogenes* was universally susceptible to Penicillin.

The second most common organism isolated was *Staphylococcus aureus* (31.9%). Most (88.0%) *Staphylococcus aureus* isolates were resistant to penicillins. Susceptibility to erythromycin and flucloxacillin was 89.8% and 88.6% respectively. Methicillin-resistant *Staphylococcus aureus* (MRSA) was cultured in 8 (3.4%) cases.

The ethnic distribution of patients affected by these two organisms and the antibiotic susceptibility results of *Staphylococcus aureus* to three common antibiotics (penicillin, erythromycin and flucloxacillin) are presented in Table 5. Although a higher percentage of Pacific people cultured *Streptococcus pyogenes* and *Staphylococcus aureus*, these ethnic differences were not statistically significant.

**Table 5:** **Distribution of *Streptococcus pyogenes *and *Staphylococcus aureus *(SA) and antibiotic susceptibility pattern of SA by ethnicity**

	**European**		**Maori**		**Pacific**		**Other**		**Total**		**p-value**
	**No.**	**%**	**No.**	**%**	**No.**	**%**	**No.**	**%**	**No.**	**%**	
*Streptococcus pyogenes*	48 (n=117)	41.0	18 (n=45)	40.0	28 (n=59)	47.5	4 (n=17)	23.5	98 (n=238)	41.2	0.366
*Staphylococcus aureus (SA)*	33 (n=117)	28.2	12 (n=45)	26.7	25 (n=59)	42.4	6 (n=17)	35.3	76 (n=238)	31.9	0.224
*SA* susceptible to Penicillin	5 (n=32)	15.6	1 (n=12)	8.3	3 (n=23)	13.0	0 (n=6)	0.0	9 (n=73)	12.3	0.716
*SA* susceptible to Erythromycin	23 (n=29)	79.3	12 (n=12)	100.0	21 (n=22)	95.5	6 (n=6)	100.0	62 (n=69)	89.9	0.097
*SA* susceptible to Flucloxacillin	29 (n=31)	93.5	10 (n=11)	90.9	17 (n=22)	77.3	6 (n=6)	100.0	62 (n=70)	88.6	0.224

### Complication, specific intervention and outcome

Many NF patients developed several complications and underwent specific interventions during their hospital stay. Table 6 shows the frequency and ethnic distribution of some complications (renal failure, shock, multi-organ dysfunction, coagulation abnormalities and myocardial infarction), specific interventions (surgical debridement, skin grafting, blood product transfusion, ICU admission and dialysis) and two specific outcomes (amputation and death). The ethnic differences in the extent of complications, interventions and outcomes were not statistically significant.

**Table 6:** Complications, specific interventions and outcomes of NF admission by ethnicity in reviewed patients

	**European (n=122)**		**Maori (n=47)**		**Pacific (n=60)**		**Other (n=17)**		**Total (n=247)**		**p-value**
	**No.**	**%**	**No.**	**%**	**No**	**%**	**No.**	**%**	**No.**	**%**	
Renal failure	54	44.3	20	42.6	24	40.0	7	41.2	105	42.2	0.957
Shock	54	44.3	17	36.2	24	40.0	9	52.9	105	42.5	0.607
Multi-organ dysfunction	21	17.2	8	17.0	12	20.0	3	17.6	44	17.8	0.970
Coagulation abnormality	31	25.4	8	17.0	11	18.3	3	17.6	53	21.5	0.539
Myocardial infarction	5	4.1	1	2.1	4	6.7	1	5.9	11	4.5	0.705
ICU admission	73	59.8	20	42.6	35	58.3	10	58.8	138	55.9	0.225
Surgical debridement	109	89.3	44	93.6	53	88.3	16	94.1	223	90.3	0.739
Skin grafting	64	52.5	26	55.3	34	56.7	7	41.2	131	53.0	0.708
Blood product transfusion	64	52.5	17	36.2	25	41.7	9	52.9	115	46.6	0.203
Dialysis	8	6.6	5	10.6	7	11.7	1	5.9	21	8.5	0.618
Amputation	22	18.0	5	10.6	5	8.3	2	11.8	34	13.8	0.285
Death	33	27.0	8	17.0	12	20.0	5	29.4	58	23.5	0.443

This is the largest NF case-series so far reported and shows several important features of the risk factors, microbiology, and outcomes for this infection. Our analysis confirms the earlier finding of a positive association of NF with increasing age and with male sex [3]. More than 80% of patients had one or more recognized predisposing conditions. For Europeans and people of Other ethnicity, two common conditions were diabetes and NSAID use, while those for Maori and Pacific people were diabetes and obesity. Increasing rates of diabetes and obesity in Pacific people and Maori are well documented [28], and these trends could partly explain the increasing incidence of NF in recent years [3]. An association has been documented between NF and NSAIDs and there are plausible mechanisms by which NSAIDs might predispose to severe bacterial infection [14]. We found that nearly a quarter of patients had a history of NSAID use within the seven days preceding onset of symptoms. This finding is consistent with the results of two studies from Middlemore Hospital in Auckland [9,12]. However, the definitive answer regarding the question of causation has not been resolved [14,29]. As in other studies [16], we found that accidental trauma, surgery, ulcer, burn and intra venous drug use (IVDU) had triggered the process in some cases. A third of patients had an antecedent history of accidental trauma (including blunt trauma without a breach in the skin).

Traditional Samoan tattooing was documented as the precipitating event for NF in four Samoan men, one of whom died. There were no further details of the process of tattooing or infection control procedures recorded in the patient notes, so we cannot implicate any particular aspect of the procedure as contributing to the risk of developing the condition. It was reported in two charts that NF developed at the site of acupuncture. No further details were available. Although there was considerable media attention and publicity about the risks from traditional Samoan tattooing, this review found that the contribution from this exposure was fortunately small in relation to the overall burden of NF. This finding is consistent with the international literature which has reported only a few sporadic cases of NF following traditional tattooing [17,30]. It is probable that some people developed less severe forms of infection (e.g. cellulitis) following traditional tattooing [20]. However, exploration of those outcomes was not part of this study.

We found that *Streptococcus pyogenes*, either singly or in combination with a staphylococcus or other organism, was the most frequent organism isolated in NF cases. Fifty-eight percent of culture positive cases in this group grew *Streptococcus pyogenes* or a non-Group A streptococcus. This finding is consistent with the microbiology findings of a case series of 198 necrotizing soft tissue infections (NSTI) in the US, where a streptococcus was isolated in 56% of cases [22]. *Staphylococcus aureus* including MRSA was cultured in more than a third of our reviewed cases for whom a microbiology result was available. This proportion is higher than that reported for the other series [9,10,12,16,22]. Only about 10% of staphylococci were MRSA (8 out of 76). A wide variation of the proportion of MRSA from NF patients has been reported [22,31-33]. Our result is near the lower end of this range [22,33].

In addition to the above two major organisms, many others were cultured from reviewed NF patients. This result is consistent with the findings of the NSTI case series already mentioned, though the frequencies were different [22].

Proportions of Pacific people with *Streptococcus pyogenes* and *Staphylococcus aureus* positive culture are higher (though not significantly) than in all other ethnic groups. From this limited information, it appears that staphylococcus may be playing a bigger role in the pathogenesis of NF in NZ, particularly affecting Pacific people. This observation might partly explain the higher rates observed in Pacific people but needs further exploration to assess whether it is an important finding.

Our study documents the severe consequences of NF in terms of the high proportion with serious complications and death. We could locate only one other study reporting the extent of health care resource used to manage NF [23]. Although some studies reported the proportion of NF cases resulting in death, limb loss and ICU care, we could not locate any large study which reported complication rates and specific interventions to manage these as reported in this current paper.

Our study has important limitations. The NF patient records reviewed were confined to those admitted to a sample of NZ hospitals from 2000 to 2006, though it did include the majority of NF cases discharged over that seven year period. Consequently, it may not be possible to generalize our findings to all hospitals in NZ and to other time periods. This study was also limited to reviewing data on NF cases and did not include a comparison population. As a result, it is difficult to evaluate the significance of the risk factors which appeared to be associated with NF, such as diabetes and NSAID use. Our review of cases was limited to information recorded in patient notes, so is likely to underestimate the proportions of some characteristics, particularly predisposing conditions and precipitating events.

## Conclusions

From the retrospective chart review results, we conclude that NF incidence and distribution is strongly influenced by the presence of various known predisposing conditions which compromise immunity, particularly in the elderly. Traditional Samoan tattooing and other body piercing procedures are not major causes. There is also potentially a contribution from changes in the virulence of the causative organisms, though we could not assess this potential factor with the data that were available.

The rising incidence of NF in NZ [3] also needs to be put in the wider context of a well documented increase in serious skin infections in children that is caused by similar bacteria [34]. The incidence of serious infectious diseases in general has risen significantly in NZ over the 20 year period from 1989 to 2008, as have ethnic and socio-economic inequalities [35]. These wider changes suggest that there may also be a contribution to NF from broad health determinants, such as disparities in income, housing conditions, and access to health services.

Given the rising incidence of NF in NZ and its severity, greater effort should be put into preventing this disease and improving the outcomes for those infected. Findings from this study suggest that high risk groups identified here should be the focus of attention. Patients over 50 years of age with important predisposing conditions (diabetes, obesity) should be alerted to this risk. In particular, they should take care to protect themselves from skin trauma and ulcers, and also ensure rapid assessment of skin infections if they develop. Further analytic studies (probably case–control) should be considered to quantify the importance of the risk factors identified here, particularly the role of pharmaceuticals such as NSAIDs, steroids, and other immune suppressive drugs.

## Abbreviations

COPD: Chronic obstructive pulmonary disease; GAS: Group A streptococcus; ICD: International Classification of Diseases; ICU: Intensive care unit; IVDU: Intravenous drug use; MRSA: Methicillin-resistant *Staphylococcus aureus*; NF: Necrotizing fasciitis; NHI: National Health Index; NSAID: Non-steroidal anti-inflammatory drugs; NSTI: Necrotizing soft tissue infections; NZ: New Zealand; US: United States.

## Competing interests

All the authors declare that that they have no competing interests.

## Authors’ contributions

DD did the literature review, conducted the hospital chart review and analysis of resulting data, interpreted data, drafted the initial study report and wrote the final report. MB conceptualized the study and formulated the study design, and contributed to data interpretation. He revised and edited the manuscript. KV did the statistical analysis and contributed to data interpretation. He revised the manuscript. All authors read and approved the final manuscript.

## Pre-publication history

The pre-publication history for this paper can be accessed here:

http://www.biomedcentral.com/1471-2334/12/348/prepub
